# Uranium and neptunium retention mechanisms in *Gallionella ferruginea*/ferrihydrite systems for remediation purposes

**DOI:** 10.1007/s11356-020-09563-w

**Published:** 2020-06-16

**Authors:** Evelyn Krawczyk-Bärsch, Andreas C. Scheinost, André Rossberg, Katharina Müller, Frank Bok, Lotta Hallbeck, Jana Lehrich, Katja Schmeide

**Affiliations:** 1 Helmholtz-Zentrum Dresden-Rossendorf, Institute of Resource Ecology, Bautzner Landstr. 400, 01328 Dresden, Germany; 2 The Rossendorf Beamline, ESRF, F-38043 Grenoble, France; 3 Microbial Analytics Sweden AB (MICANS), SE-43535 Mölnlycke, Sweden

**Keywords:** Actinides, Sorption, Microorganism, Bacteriogenic iron oxyhydroxides, XAS, ATR FT-IR spectroscopy

## Abstract

The ubiquitous β-Proteobacterium *Gallionella ferruginea* is known as stalk-forming, microaerophilic iron(II) oxidizer, which rapidly produces iron oxyhydroxide precipitates. Uranium and neptunium sorption on the resulting intermixes of *G. ferruginea* cells, stalks, extracellular exudates, and precipitated iron oxyhydroxides (BIOS) was compared to sorption to abiotically formed iron oxides and oxyhydroxides. The results show a high sorption capacity of BIOS towards radionuclides at circumneutral pH values with an apparent bulk distribution coefficient (*K*_d_) of 1.23 × 10^4^ L kg^−1^ for uranium and 3.07 × 10^5^ L kg^−1^ for neptunium. The spectroscopic approach by X-ray absorption spectroscopy (XAS) and ATR FT-IR spectroscopy, which was applied on BIOS samples, showed the formation of inner-sphere complexes. The structural data obtained at the uranium L_III_-edge and the neptunium L_III_-edge indicate the formation of bidentate edge-sharing surface complexes, which are known as the main sorption species on abiotic ferrihydrite. Since the rate of iron precipitation in *G. ferruginea*-dominated systems is 60 times faster than in abiotic systems, more ferrihydrite will be available for immobilization processes of heavy metals and radionuclides in contaminated environments and even in the far-field of high-level nuclear waste repositories.

## Introduction

It is well known that in many freshwater and marine environments, where the oxygen concentrations can be very low (e.g., at anoxic-oxic transition zones), microaerophilic iron(II) oxidizers are common (Emerson and Moyer 1997; Edwards et al. 2003). Whereas abiotic iron oxidation is retarded at low oxygen concentrations, certain specialized species of iron oxidizers (e.g., *Gallionell*a spp.) are able to grow (Edwards et al. 2003), thus contributing to iron oxidation. Phylogenetically, these bacteria belong to the phylum Proteobacteria, which includes the freshwater genera *Leptothrix* (Fleming et al. 2011), *Gallionella* (Kucera and Wolfe 1957), and *Sideroxydans* (Weiss et al. 2007) as well as the marine genus *Mariprofundus* (Singer et al. 2011; Melton et al. 2014). Microbial iron(II) oxidation by microaerophilic iron(II) oxidizers at low oxygen concentrations occurs according to the following stoichiometric equation (Melton et al. 2014):

1 $$ {4\mathrm{Fe}}^{2+}+10{\mathrm{H}}_2\mathrm{O}+{\mathrm{O}}_2\to 4\mathrm{Fe}{\left(\mathrm{OH}\right)}_3+{8\mathrm{H}}^{+} $$

The reaction is similar to the physico-chemical reaction in abiotic systems (Ankrah and Søgaard 2009). Decisive factors as to whether the precipitation of iron oxyhydroxide is going to be biotic or abiotic are redox potential and pH value. The iron(II)-oxidizing bacterium *Gallionella*, for example, requires a lower pH and a lower redox potential than commonly necessary for abiotic formation of iron oxyhydroxide (Hanert 1992; Søgaard et al. 2000). By thermodynamic analysis of the electrochemical equilibrium, the theoretical boundary between the field of ferrous solubility and the formation of biogenic ferric precipitates was defined by Ankrah and Søgaard (2009). Ferric iron precipitates rapidly due to the low solubility of ferric iron compounds in the area characterized by a pH range from 5.5–8.0 with a redox potential limit, moving from + 420 to + 50 mV. Eggerichs et al. (2014) defined the ferrous iron oxidation by *Gallionella*, which increases with increasing ferrous iron concentrations, showing a Michaelis-Menten-like distribution with K_Fe(II)_ = 2.61 ± 1.67 mg L^−1^, which is typical for iron-rich natural waters. In their studies about pH dependencies, the authors conclude that compared to abiotic oxidation, iron oxidation by *Gallionella* occurs within a much broader pH/oxygen range. There are different explanations for the bacteriogenically induced oxidation of ferrous iron. After Banfield et al. (2000), bacteria such as the *Gallionella* spp. and the *Leptothrix* spp. are able to oxidize enzymatically dissolved ferrous iron. Melton et al. (2014) assumed that the iron(II) oxidation may be carried out by an outer cell membrane iron(II)-oxidizing protein to prevent ferric iron mineral precipitation inside the cell. After Søgaard et al. (2000), the precipitation takes place in contact with the exopolymers, which act as catalysts for the oxidation-precipitation process of iron. This assumption is supported by the fact that biogenic ferric iron has a much higher density than physico-chemically precipitated colloidal ferrihydrite. Søgaard et al. (2000) assume that this phenomenon can be attributed to the fact that biogenically precipitated ferric iron is in contact with exopolymers. Soluble ferrous iron either adsorbs directly on the surface of exopolymers, where it becomes oxidized, or is attached to the exoploymers of *Gallionella ferruginea* in a colloidal form (Ankrah and Søgaard 2009). Thus, iron is oxidized and precipitates with a rate about 60 times faster than that in the abiotic process. The faster kinetic indicates a catalytic activity due to the presence of exopolymers from *G. ferruginea* (Søgaard et al. 2000), causing a rapid oxidation and formation of insoluble ferric hydroxides (Ankrah and Søgaard 2009). In a recent study, Hallbeck and Pedersen (2014) mentioned that whether the iron oxidation on the stalk is enzymatic or whether the stalk acts as a surface catalyst for the oxidation reaction is not known. Most of the 2- to 3-nm diameter-formed particles are attached to the surrounding negatively charged polymers (e.g., *Gallionella* stalks) or are flocculated to form colloidal aggregates (Banfield et al. 2000) and coatings on other mineral particles.

The minerals formed include two-line ferrihydrite, a nano-scale ferric iron oxyhydroxide mineral with the formula of Fe_10_O_14_(OH)_2_ (Melton et al. 2014), lepidocrocite (Chan et al. 2011), and akageneite (Chan et al. 2004). These mineral phases are characterized by large surfaces, which are known to provide active adsorption sites for inorganic and organic contaminants. Iron oxyhydroxide minerals in general are found ubiquitously in the environment, where high concentrations of ferric iron are produced by either chemical oxidation or bacterial oxidation of ferrous iron (Ferris et al. 1989), e.g., in soils; weathered rocks; sediments; and water column of lakes, rivers, and oceans. Moreover, they are also present in contaminated soils and groundwater, resulting, e.g., from mining and milling activities, engineered nuclear waste environments, and nuclear decommissioning, where heavy metals and radionuclides were already released into the environment, thus posing a danger to humans and animals due to an ultimate potential incorporation into the food chain. Some authors (Pedersen and Karlsson 1995; Pedersen 1997; Ferris et al. 1999; Hallbeck and Pedersen 2005; Anderson et al. 2006) described biofilms formed in open fracture zones and on tunnel walls at the Äspö Hard Rock Laboratory (HRL) in the south-east of Sweden, where groundwater, including biological materials, is seeping in. The biofilms are predominantly formed by the indigenous iron-oxidizing bacterium *Gallionella* with up to 90 wt% precipitated ferric oxyhydroxide, thus providing an abundant surface area with remarkable highly reactive surface sites (Ferris et al. 1989; Nelson et al. 1995; Ferris et al. 2000; Anderson and Pedersen 2003). As a consequence, the biofilms can act as dominant sorbents for significant amounts of dissolved metals under certain environmental conditions, e.g., redox potential and pH (Ferris et al. 1989; Nelson et al. 1995). At near-neutral pH, the biogenically produced iron oxyhydroxides have the potential to adsorb 30–15,000 times more lanthanides than abiotic and synthetic iron oxyhydroxides (Anderson and Pedersen 2003). They may be more reactive sorbents also for contaminants, such as arsenic and chromium as recent studies showed. The sorption of arsenic(V) to biogenically produced iron oxyhydroxides was approximately three times higher than what was observed for synthetic 2-line ferrihydrite (Sowers et al. 2017). Whitaker et al. (2018) mentioned that biogenically produced iron oxyhydroxides may enhance chromium(VI) adsorption and even reduction. The authors attributed the higher adsorption property to the poorly ordered structures, large surface areas, and incorporation of cell-derived organic matter. The Äspö HRL is of particular interest for researchers, since the facility serves as an analog for a final nuclear waste repository for high-level radioactive waste. In the event of ingress of water, bacteriogenically produced iron oxyhydroxides may offer an additional barrier to the migration of dissolved radionuclides, which might be released from within the waste containers. Whereas a lot of previous investigations were performed to quantify metal ion sorption, there is still a lack concerning the identification of surface complexes and the determination of the structural coordination of sorption complexes on biogenically formed iron oxyhydroxides. The present study aims at responding to these points using spectroscopic methods for the molecular scale structural characterization, i.e., extended X-ray absorption fine structure (EXAFS) and in situ attenuated total reflection Fourier-transform infrared (ATR FT-IR) spectroscopy. We focused our studies in the present work on two problematic α-emitting radionuclides, uranium and neptunium, which are characterized by long half-lives (^238^U 4.47 × 10^9^ years, ^237^Np 2.14 × 10^6^ years) and high radio- and chemotoxicity. Uranium occurs in the environment as a consequence of mining activities, leaching of radioactive wastes, and as a contaminant at nuclear legacy sites. Under aerobic conditions, uranium(VI) is mobile due to its high solubility. Under anaerobic conditions, uranium is typically reduced to poorly soluble uranium(IV). In geological disposal facilities, containing spent nuclear fuel, uranium will be the most significant radionuclide by mass (Marshall et al. 2014). In case of an accident, it might be released from within the waste containers. The oxidation state, speciation, and migration or immobilization behavior of uranium and radionuclides resulting from its radioactive decay will then depend on the surrounding conditions (e.g., pH, redox potential, oxygen concentration). Neptunium is one of the most important radionuclides of concern for long-term emplacement of nuclear waste due to its long half-life. The concentration of ^237^neptunium will increase with time due to the radioactive decay of ^241^plutonium and ^241^americium (Kaszuba and Runde 1999; Lloyd et al. 2000). In oxic environments, aqueous neptunium speciation is dominated by the pentavalent cation, NpO_2_^+^ in the pH range 5 to 8 (Girvin et al. 1991) and at redox potentials between 100 and about 800 mV (Lieser and Mühlenweg 1988). Under anaerobic conditions neptunium(V) is reduced to the tetravalent form as sparingly soluble Np(OH)_4_.

In the present study a *G. ferruginea* strain, isolated from a 60-m deep, unlined drinking-water well, was cultured under microaerophilic conditions to obtain biogenically formed iron oxyhydroxide precipitates. The formed bacteriogenic iron oxyhydroxides (BIOS), an intermix of *G. ferruginea* cells, stalks, extracellular exudates, and precipitated iron oxyhydroxides, was used for batch sorption experiments with uranium(VI) and neptunium(V) under anaerobic conditions. X-ray absorption spectroscopy (XAS) and ATR FT-IR were applied to identify surface complexes and their structural coordination on biogenically formed iron oxyhydroxides. The results are compared with abiotic systems to demonstrate that microaerophilic iron(II) oxidizers contribute strongly to the immobilization of radionuclides.

## Materials and methods

### Characterization and cultivation of *Gallionella ferruginea*

The strain of *G. ferruginea* used in this study was isolated by Lotta Hallbeck (MICANS, Sweden) from a 60-m deep, unlined drinking-water well. The well is situated in Hindås, in the southwest of Sweden. *G. ferruginea* is a stalk-forming, autotrophic, and iron-oxidizing bacterium, which was first described by Ehrenberg (1836). It is described in detail in several papers by Hallbeck and co-authors. The phylogenetic position of *Gallionellaceae* is among the β-Proteobacteria, as determined by 16S-rDNA sequence comparisons (Hallbeck et al. 1993). The cells have a curved rod shape with a size of 1.6–2.5 × 0.5–0.8 mm (Hallbeck and Pedersen 1990). The cells are Gram-negative (Teichmann 1935) with an outer membrane and a thin peptidoglycan cell wall (Hallbeck and Pedersen 2005). *G. ferruginea* can be recognized under the microscope by its twisted stalks, which are produced when iron-rich groundwater comes in contact with air at neutral pH (Hallbeck and Pedersen 2014).

The carbon source for *G. ferruginea* is carbon dioxide, and the energy and electron source is ferrous iron, which is oxidized to ferric iron with oxygen as electron acceptor. The culture of *G. ferruginea* strain, provided by MICANS, was cultivated in our laboratory. A mineral salt solution (MSS) was prepared as described by Kucera and Wolfe (1957), modified by Hanert (1981), and briefly described in Hallbeck and Pedersen (1990), consisting of 1 g NH_4_Cl, 0.4 g MgSO_4_ × 7H_2_O, 0.1 g CaCl_2_ × 2H_2_O, 0.5 g K_2_HPO_4_, and 1 L Milli-Q water. The MSS medium was autoclaved and cooled to 5 °C. Several 300 mL Erlenmeyer flasks with around 150 mL MSS medium were infused with sterile-filtered CO_2_ for 30 s to reach pH 4.6−4.8. After the CO_2_ infusion, 600 screw-capped glass tubes (180 × 16 mm), used as culture vessels, were filled each with 10 mL MSS. Iron sulfide was prepared as described by Hanert (1981) by dissolving 7.8 g FeSO_4_(N_6_H_4_)_2_SO_4_ (Mohr’s salt) and 4.8 g Na_2_S separately, each in 200 mL boiling Milli-Q water, and subsequently pouring the ferrous iron solution into the Na_2_S solution. Glass Pasteur pipettes were used to add 20 drops of sterile iron sulfide solution carefully by inserting the pipette to the bottom of the tubes. When the iron sulfide had settled, the tubes were immediately inoculated with *G. ferruginea* culture. The culture was incubated at a temperature below 25 °C due to the heat sensitivity of this strain of *G. ferruginea*. The cultures were sub-cultured once a week.

### Precipitation of ferric oxyhydroxide

**Fig. 1:**
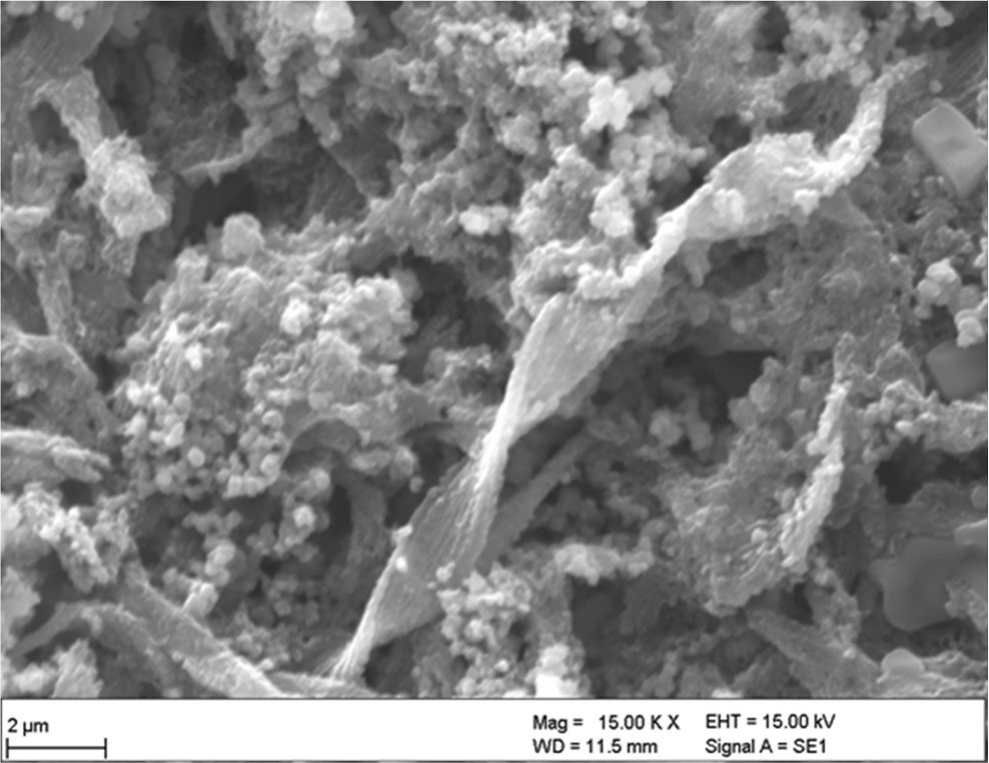
SEM image of BIOS, as a mixture of G. ferruginea cells and stalks, extracellular exudates, and ferric oxyhydroxide

Already within the first days of *G. ferruginea* cultivation in the tubes, a yellowish coloration was visible in the solution, indicating that ferrous iron is oxidized by the microaerophilic ferrous oxidizer *G. ferruginea* at the currently available low O_2_ concentrations. As described by numerous authors (e.g., Hanert 1981; Hallbeck and Pedersen 1995; Stumm and Morgan 1996; Ferris et al. 1999; Banfield et al. 2000; Melton et al. 2014), the bacteriogenic-induced oxidation of Fe(II) leads often to a precipitation and encrustation on the twisted stalk materials of *G. ferruginea*. Iron-rich particles with 2–3 nm diameters are formed as colloids and are attached to the negatively charged polymers of *G. ferruginea* stalks as shown by using scanning electron microscopy (SEM) in Fig. 1. Since at near-neutral pH the solubility of ferric iron compounds is low, Banfield et al. (2000) suggested that a solution supersaturation immediately could take place with respect to ferrihydrite solubility. By selected area electron diffraction (SAED), the authors were able to show that the samples mostly consisted of randomly oriented two-line ferrihydrite. During the cultivation of *G. ferruginea*, high amounts of ferric oxyhydroxide were formed as small, roundish particles (0.1–0.5 μm) within a short time in our study.

### Separation of BIOS

The formed BIOS was removed from the tubes by using glass Pasteur pipettes. It was sampled in 50 mL Greiner tubes and centrifuged (Ultracentrifuge Optima XL100K, Rotor: SW 32Ti; Beckman Coulter, USA) at 187,000×*g* for 20 min. The supernatant was removed, and the BIOS was washed four times with sterile tap water. The BIOS was separated and transferred into inert gas boxes (N_2_ atmosphere) for uranium and neptunium sorption experiments, respectively.

### Sorption experiments with uranium(VI) and neptunium(V)

For uranium sorption experiments, 8.4 mg of the BIOS was mixed with 50 mL sterile tap water, where UO_2_(ClO_4_)_2_ was added prior to achieve an initial uranium(VI) concentration of 0.09 mM. In another experiment NpO_2_(ClO_4_) was added to 10 mL sterile tap water to reach a final neptunium(V) concentration of 0.09 mM and subsequently mixed with 5.6 mg BIOS. The pH was adjusted in each experiment to 7.8 by adding diluted NaOH solution. The suspensions were shaken on a horizontal shaker for 183 h and 185 h, respectively, at room temperature. At the beginning of the uranium(VI) sorption experiment, aliquots containing 0.5 mL of the suspension were taken every 0.5 h, since sorption of uranium(VI) on abiotic ferrihydrite is known to be very fast (Waite et al. 1994). The samples were centrifuged at 187,000×*g*, and the uranium(VI) concentration in the supernatants was determined by means of inductively coupled plasma mass spectrometry (ICP-MS) using an ELAN 9000 ICP-MS spectrometer (PerkinElmer, Überlingen, Germany) after acidification. The neptunium(V) concentration was determined by liquid scintillation counting (LSC, Winspectral α/β, Wallac 1414, Perkin Elmer, Rodgau, Germany) using α-β discrimination. For this, 100 μL of the centrifuged aliquot was taken and mixed with 5 mL Ultima Gold™ scintillation cocktail (Perkin Elmer). During the experiments, redox potentials and pH values were recorded. The pH values were measured with a conventional pH electrode (WTW SenTix 20, Germany), regularly calibrated by means of commercial buffer solutions. Redox potentials of the sample solutions were measured using a combined platinum Ag/AgCl redox electrode (WTW Electrode SenTix ORP, Germany). The data are reported as *E*_h_ values, i.e., as potentials against the standard hydrogen electrode. For reproducibility, the sorption experiments were performed in duplicate.

### Thermodynamic calculation

The chemical composition of the supernatants from the uranium and neptunium sorption experiments was determined at the end of the experiments. The data were used to calculate the predominance fields of various uranium and neptunium species, respectively, at room temperature. The corresponding pH–*E*_h_ diagrams were constructed using the geochemical speciation code Geochemist’s Workbench, version 12.0.4/Act2. For uranium, the database used was the thermo.dat accompanying the code, supplemented by the most recent NEA database for aqueous uranium species (Guillaumont et al. 2003) and by thermodynamic data of the aqueous species Ca_2_UO_2_(CO_3_)_3(aq_) (Bernhard et al. 2001). This species is the major species in the area characterized by a higher pH (6.8–10.9) and a higher redox potential limit, moving from + 60 mV at pH 6.8 to − 250 mV at pH 10.9. For neptunium, thermodynamic data of the Lawrence Livermore National Laboratory thermodynamic database were supplemented by the most recent NEA database (Lemire et al. 2001; Guillaumont et al. 2003).

### X-ray absorption spectroscopy

After separation of the supernatants, the U-BIOS and Np-BIOS samples were filled as wet paste into polyethylene sample holders under anoxic conditions in a glove box. The sample holders were hot sealed and immediately shock frozen in liquid N_2_. The samples were transported in a special dewar filled with liquid N_2_ (Voyageur 12, Air Liquide Deutschland GmbH, Germany) to be analyzed at the Rossendorf Beamline of the European Synchrotron Radiation Facility (ESRF) in Grenoble (France) by XAS. XAS is a powerful method to provide detailed element-specific information on the speciation and local coordination environment of the probed element. It includes X-ray absorption near-edge structure (XANES) spectroscopy for detailed element-specific information on the oxidation state and site geometry through interpreting both the position and the structure of the absorption edge as well as extended X-ray absorption fine structure (EXAFS) (~ 50–1000 eV above the edge), which is used to extract detailed information on local coordination and bonding environment. It is an element-specific bulk method, giving information about the average, local structural, and compositional environment of the X-ray-absorbing atom. U L_III_- and Np L_III_-edge spectra of frozen pellets were collected in fluorescence mode using a 13-element germanium detector and a helium cryostat at 15 K. During fluorescence measurements, eight to ten scans were recorded for each sample and then averaged. The energy scale was calibrated using the maximum of the first derivative of the K-edge absorption spectrum of yttrium (17,038 eV). The EXAFS spectra were analyzed according to standard procedures including statistical weighting of the 13 fluorescence channels and dead-time correction using WinXAS 3.2 (Ressler 1998). In addition, the spectra were investigated with the Iterative Transformation Factor Analysis (ITFA) software package (Rossberg et al. 2003).

### In situ attenuated total reflection Fourier-transform infrared spectroscopy

In situ ATR FT-IR spectroscopy with a sub-minute time resolution was performed for monitoring and complementary molecular identification of the neptunium(V) sorption process on the BIOS. Infrared spectra were measured from 1600 to 750 cm^−1^ on a Bruker Vertex 80/v vacuum spectrometer equipped with a Mercury Cadmium Telluride detector. Spectral resolution was 4 cm^−1^, and spectra were averaged over 256 scans. A horizontal diamond crystal with nine internal reflections (DURA SamplIR II, Smiths Inc.) was used. Further details on the experimental ATR FT-IR spectroscopy setup are compiled in Müller et al. (2012). The performance of in situ sorption experiments requires a thin sample film prepared directly on the surface of the ATR diamond crystal as stationary phase. This was accomplished by pipetting 5 μL of the sample on the ATR crystal and subsequent drying with a gentle stream of N_2_. Flow-through measurements at a rate of 0.1 mL min^−1^ were performed using a flow cell (*V* = 200 μL). First, the film was flushed with a blank solution (sterilized tap water, pH 8) for 60 min conditioning. In a second step, a 50 μM neptunium(V) solution (sterilized tap water, pH 5) was rinsed for sorption during the next 60 min. Finally, the neptunium(V)-loaded film was flushed again with the blank solution (60 min) in order to gain information on the reversibility of the sorbed species.

The applied principle of reaction-induced difference spectroscopy allows the detection of very small absorption changes provoked by the sorption process in comparison with the very strong absorbing background, i.e., water and sample film. Further details on the calculation of difference spectra are given in Müller et al. (2012) and Müller et al. (2013).

## Results and discussion

### Uranium and neptunium sorption

**Fig. 2:**
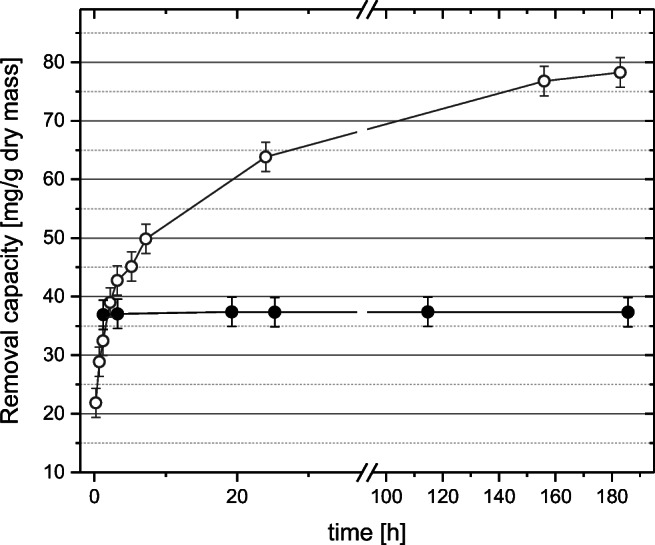
Uranium (○) and neptunium (●) removal capacity of BIOS as a function of time at pH 7.8 and room temperature

In our anaerobic experiments, uranium(VI) and neptunium(V) were added aseptically from an actinide stock solution to the BIOS suspension to reach an actinide concentration of 0.09 mM at a pH of 7.8. At the end of the experiments, the uranium concentration in the BIOS suspension was determined to have declined to 0.03 mM, clearly indicating that uranium had been removed from solution and immobilized on the BIOS sample. As shown in Fig. 2, most of the uranium sorption occurred during the first 24 h of the experiment, indicating a rapid initial sorption process, which is also typical for the binding of inorganic ions to abiotic ferrihydrite (Waite et al. 1994). After 183 h, a uranium uptake of 78-mg U g^−1^ dry mass was determined. An apparent bulk distribution coefficient (*K*_d_) of 1.23 × 10^4^ L kg^−1^ was calculated following the conventional operational definition according to Bau (1999) as the ratio between sorbed [U] per kg BIOS and dissolved [U] per L water.

For the neptunium sorption experiment, it was clearly shown that already in the first hour, neptunium was almost completely removed from the solution, which led to an uptake of 37 mg Np g^−1^ dry mass under the applied experimental conditions. The calculated apparent bulk distribution coefficient (*K*_d_) amounts to 3.07 × 10^5^ L kg^−1^. The determined *K*_d_ values for uranium and neptunium result from the fact that biogenic iron oxyhydroxide samples in general consist of inorganic and organic material, where the organic material (e.g., bacteria, stalks) and their deprotonated carboxylate and phosphate functional groups provide active surface sites (Warren and Zimmerman 1994; Ferris et al. 1989). After Ferris et al. (1999), the *K*_d_ values consequently decrease with increasing iron oxyhydroxide content and decreasing content of organic material. Recent studies suggest that the higher sorption capacity of heavy metals results can be attributed to the effect of the physical and chemical characteristics of organics in BIOS (Field et al. 2019). Kikuchi et al. (2019) assume that the negatively charged BIOS are more electrostatically favorable for the adsorption of cations, such as Cs(I), than the positively charged abiogenic ferrihydrite.

### Thermodynamic calculation

**Fig. 3:**
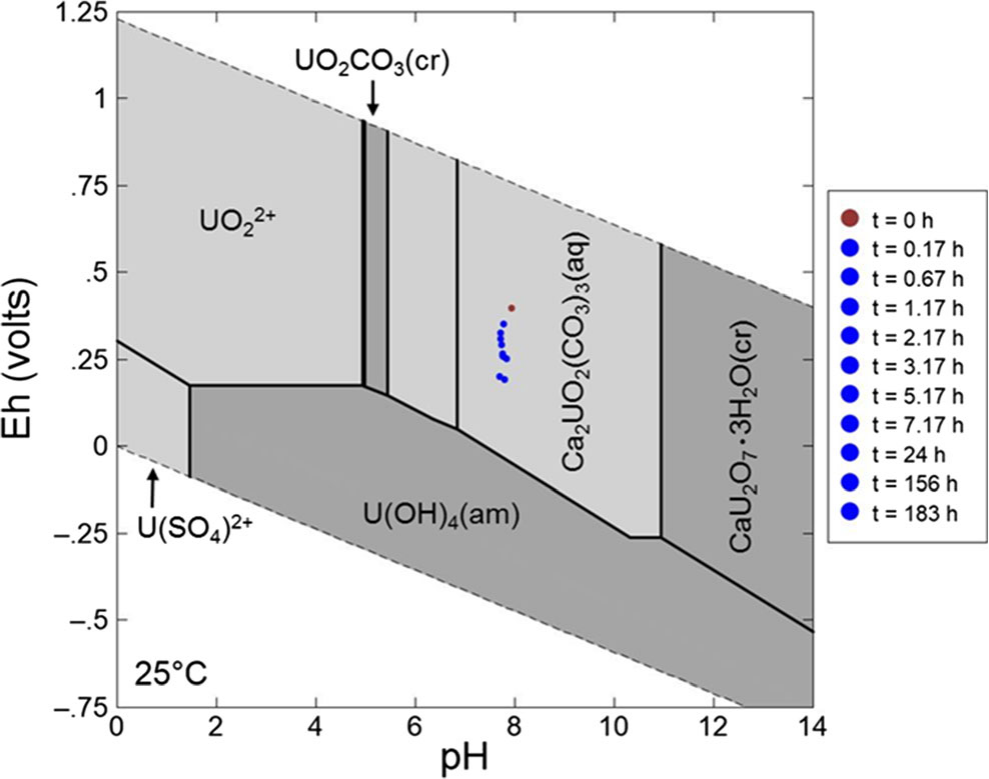
pH–*E*_h_ predominance diagram for uranium species (0.09 mM uranium, at room temperature) after thermodynamic calculation using the geochemical speciation code Geochemist’s Workbench Version 10.0.5/Act2 and the analytical data of the supernatant, determined at the end of the experiment. The plotted pH and *E*_h_ data correspond to the measured values in the supernatant at different times during the experiment

**Fig. 4:**
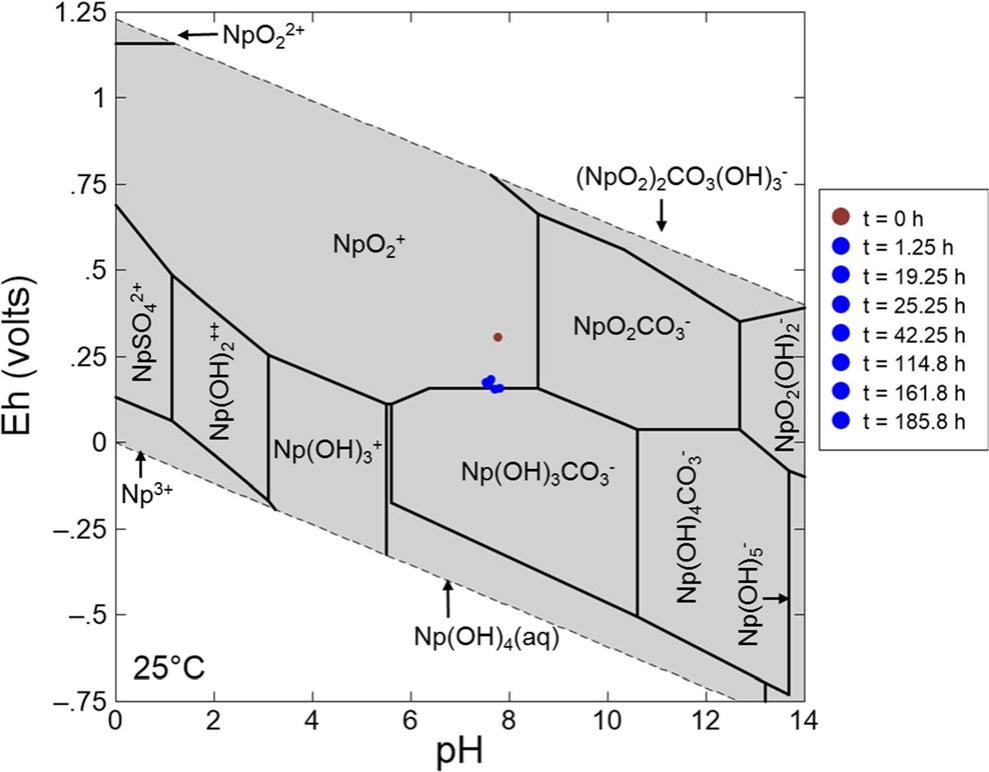
pH–*E*_h_ predominance diagram for neptunium species (0.09 mM neptunium, at room temperature) after thermodynamic calculation using the geochemical speciation code Geochemist’s Workbench Version 10.0.5/Act2 and the analytical data of the supernatant, determined at the end of the experiment. The plotted pH and *E*_h_ data correspond to the measured values in the supernatant at different times during the experiment

When plotting the measured pH and *E*_h_ values of the supernatant, which decreased from the beginning to the end of the uranium sorption experiment (pH 7.78 to 7.38 and *E*_h_ 395 ± 30 mV to 174 ± 30 mV, respectively), the predominance of an aqueous calcium uranyl carbonate species is predicted (Fig. 3), showing that uranium clearly exists in the uranium(VI) stability field. The Ca_2_UO_2_(CO_3_)_3(aq)_ species is characterized by a low sorption affinity to minerals as was shown, for instance, for ferrihydrite and quartz (Fox et al. 2006), clay (Joseph et al. 2013; Philipp et al. 2019), and granite (Schmeide et al. 2014). Brooks et al. (2003) have shown that calcium uranyl carbonate complexes inhibit microbial reduction of uranium(VI) under certain conditions. Furthermore, Singer et al. (2009, 2012) found that abiotic reduction of uranium(VI) by iron(II)-bearing minerals (magnetite and chlorite) only occurs in the absence of calcium, but it is inhibited in the presence of both, calcium and carbonate. Consequently, the presence of Ca_2_UO_2_(CO_3_)_3_ species in solution is the reason for the lower *K*_d_ value determined for uranium sorption onto BIOS. For the neptunium sorption experiment, the thermodynamic calculation shows that under ambient conditions, NpO_2_^+^ is the major species in the area characterized by a wide pH range (0–8.7) with a redox potential limit, moving from + 690 at pH 0 to 120 mV at pH 5.6 and 200 mV at pH 8.7. The pH and *E*_h_ values of the supernatant, which were measured at different times during the experiment, were plotted within the diagram (Fig. 4). The results show that already in the first hours of the experiment, the *E*_h_ value decreased from 395 ± 30 mV, which is located within the neptunium(V) stability field, towards the neptunium(V)/neptunium(IV) border with an *E*_h_ value of 174 ± 30 mV. A reduction of neptunium(V) to neptunium(IV), which would lead to an increased neptunium retention, cannot be excluded completely based on these thermodynamic data, but could be excluded by XANES (cf. paragraph 3.4. below).

### In situ ATR FT-IR

**Fig. 5:**
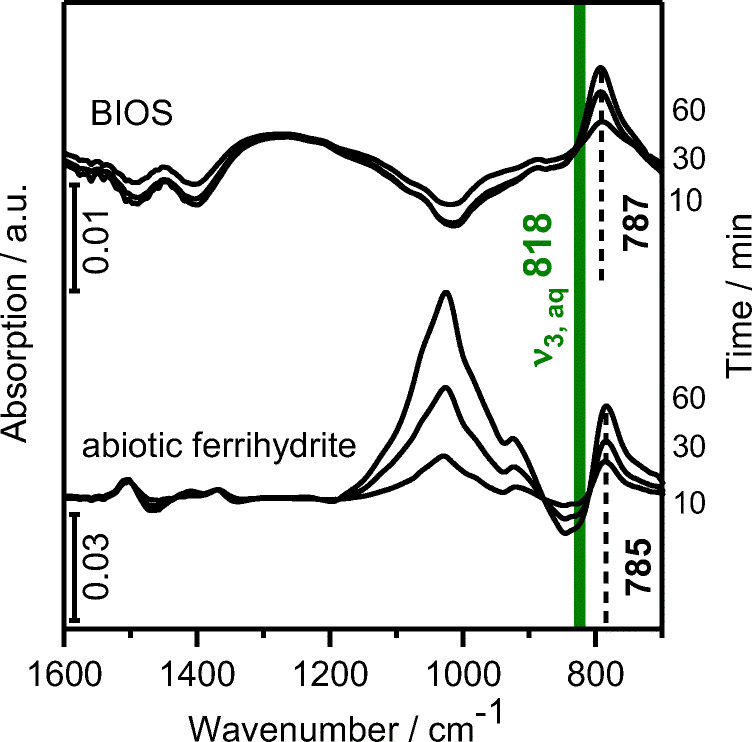
In situ time-resolved mid-IR spectra of Np(V) sorption on BIOS and abiotic ferrihydrite (50 μM initial Np(V), sterilized tap water, pH 8, flow rate 0.1 mL min^−1^). The spectra of the sorption process are recorded at various times after induction, as given. Ordinate scaling is given by the bar in units of optical density. Other values indicated are in cm^–1^

The BIOS films prepared on the ATR crystal were flushed with a blank solution at the same conditions as used in the sorption experiment. The resulting spectra after 60 min of conditioning provided no spectral features, indicating that a steady state was obtained (data not shown). The accumulation of neptunium(V) on the BIOS surface can directly be observed by the increasing band below 800 cm^−1^ upon sorption (Fig. 5). It can be assigned to the antisymmetric stretching vibrational mode v_3_ of NpO_2_^+^. The increasing signal and the very good signal-to-noise ratio indicate the sorption of neptunium(V). A solution species could hardly be detected at the neptunium(V) concentration of 50 μM. In aqueous solution at similar conditions, the free NpO_2_^+^ is present. This species shows IR absorption at 818 cm^−1^ as indicated in Fig. 5 by the green line. The shift by 31 cm^−1^ in the case of BIOS indicates a strong binding of neptunium(V) to the substrate. The same is valid for abiotic ferrihydrite as shown in Fig. 5, as well as for hematite (Müller et al. 2015). The different intensities may be due to different film heights prepared on the ATR crystal. The band arising at 1050 cm^−1^ is probably due to arrangements of surface functionalities. The reversibility of the sorption process, monitored by a flushing step with the blank solution (data not shown), showed a very small negative band at the same frequency as the band arising during sorption, i.e., at 787 cm^−1^. This indicates that minor parts of the sorbed species are removed from the interface under the experimental conditions.

### Uranium(VI) and neptunium(V) coordination environment studied with XAS

**Fig. 6:**
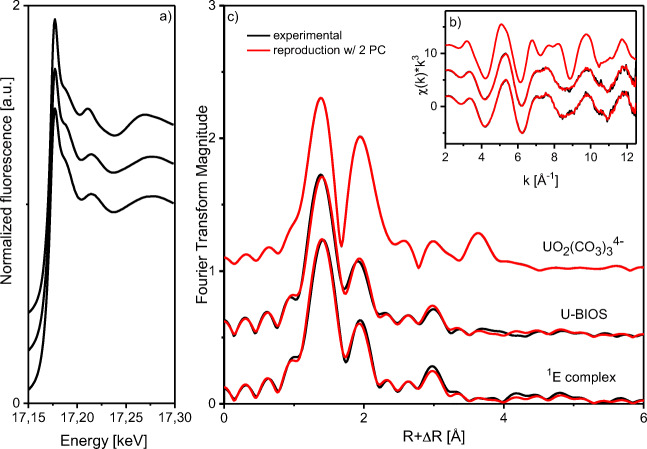
Uranium L_III_-edge XANES spectra (**a**) of the aqueous tricarbonato complex and uranyl sorption complex (^1^E) in comparison with U-BIOS sample. Uranium L_III_ k^3^-weighted EXAFS spectra (**b**) and corresponding Fourier transform (**c**) of uranium sorbed on the BIOS sample together with the endmember species observed for ferrihydrite sorption samples, i.e., a bidentate edge-sharing uranyl sorption complex (^1^E) and the aqueous tricarbonate complex (UO_2_(CO_3_)_3_^4−^) representative for a type B ternary uranyl-carbonato sorption complex taken from Rossberg et al. (2009)

**Fig. 7:**
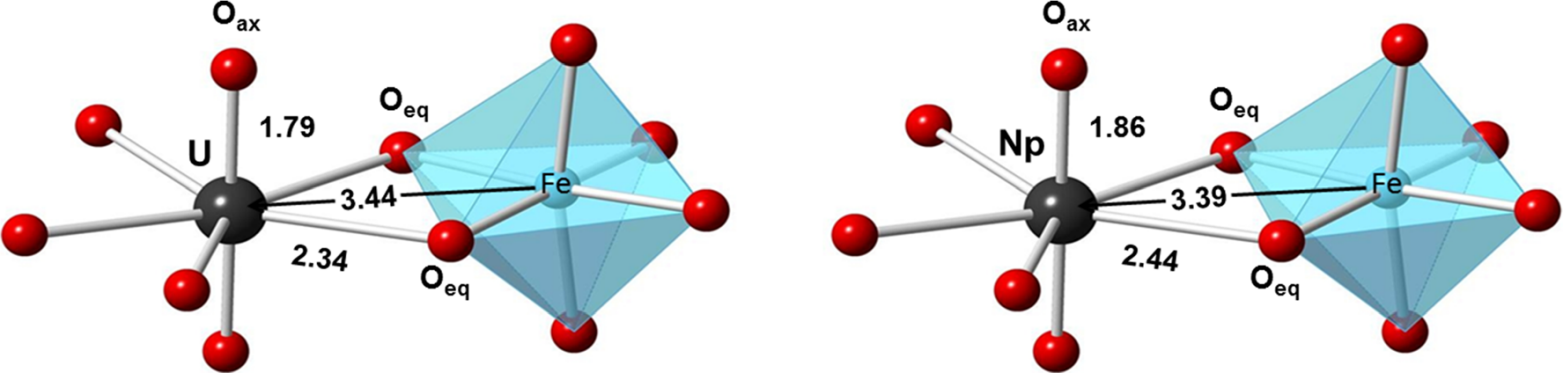
Structural models of the sorption complexes with radial distances for U-BIOS and Np-BIOS, where the UO_2_^2+^ ion or the NpO_2_^+^ ion bound via edge-sharing to one Fe(O,OH)_6_ octahedron

Direct structural data have been obtained with EXAFS at the uranium L_III_-edge and the neptunium L_III_-edge to identify the structure of uranium and neptunium species associated with the BIOS samples after the sorption experiments. In Fig. 6, the XAS spectra are shown for U-BIOS sample along with those of selected references, e.g., the bidentate edge-sharing uranyl sorption complex (^1^E) and the aqueous uranyl tricarbonate complex (UO_2_(CO_3_)_3_^4-^). The k^3^-weighted EXAFS spectrum and its Fourier-transform magnitude (Fig. 6 b and c) of the studied U-BIOS sample bears close resemblance to the bidentate edge-sharing inner-sphere sorption complex (^1^E), which is the most prominent surface species in the absence of carbonate and the main sorption species on abiotic ferrihydrite (Rossberg et al. 2009). The tricarbonato surface complex was identified by Hiemstra et al. (2009) as a major ternary species in systems equilibrated at elevated *p*CO_2_ and above-neutral pH, where high carbonate concentrations occur. Since CO_2_ was present during the cultivation of the *G. ferruginea* strain, the BIOS formed in this system may have a relatively high carbonate loading on its surface. This carbonate loading might cause the formation of a small portion of the uranyl-carbonato sorption complex. To elucidate the presence of a second sorption species, we performed iterative transformation factor analysis (ITFA) using the spectra of the ^1^E uranyl ferrihydrite sorption complex and the uranyl-carbonato sorption complex as endmember species. The good reproduction of the U-BIOS spectrum by applying only two principal components (compare the black and red lines in Fig. 6 b and c) is a proof that only two species are present and that these are represented by the two endmember species. Furthermore, we determined by using ITFA that the ^1^E complex is in fact predominant with 95%, while only 5% of the ternary uranyl-carbonato complex is present. Correspondingly, the shell fit showed only the local structure of the ^1^E complex. The XANES position and especially its fine structure as shown in Fig. 6(a) are indicative of the hexavalent uranyl moiety, as it was expected according to the thermodynamic calculation of the uranium-BIOS suspension. Hence, the spectrum shows no indication for a significant amount of uranium(IV) in the U-BIOS sample. The EXAFS structural parameters (see Table 1) obtained from the theoretical curve fit indicate the formation of a bidentate inner-sphere complex. The fit of the first and second oxygen shell of the uranium L_III_-edge EXAFS spectrum showed five equatorial (eq) oxygen atoms with an average U–O_eq_ distance of 2.34 Å and two axial (ax) oxygen atoms at a U–O_ax_ distance of 1.79 Å. The appearance of O_ax_ and O_eq_ verifies the hexavalent oxidation state of uranium. The short radial U–Fe distance of 3.44 Å indicates a bidentate edge-sharing linkage of the UO_2_^2+^ cation to an Fe(O,OH)_6_ octahedron (Fig. 7).

**Table 1:** Uranium L_III_ and Neptunium L_III_ EXAFS shell fit results of BIOS samples (S_0_^2^ = 0.9, fit range 2.0–12.5 Å^−1^)

Sample	O shell			Fe shell			∆E_0_ [eV]^d^	χ^2^ res [%] ^e^
	CN^a^	R [Å]^b^	σ^2^ [Å^2^]^c^	CN	R [Å]^b^	σ^2^ [Å^2^]^c^		
U-BIOS	2.0^f^ O 5.0 O	1.79 2.34	0.0017 0.0100	0.5	3.44	0.0080	11.9	14.6
Np-BIOS	2.0^f^ O 5.2 O	1.86 2.44	0.0016 0.0094	1.5	3.39	0.0100	13.6	13.5

^a^CN: coordination number, error ± 25%

^b^R: radial distance, error ± 0.01 Å

^c^σ^2^: Debye-Waller factor, error ± 0.0005 Å^2^

^d^∆E_0_: shift in threshold energy

^e^χ^2^ res: normalized least squares residual

^f^Parameters were fixed in the data fitting

**Fig. 8:**
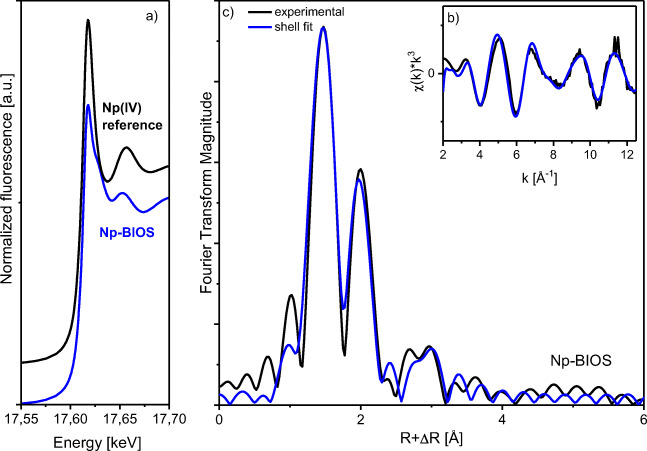
Neptunium L_III_-edge k^3^-weighted EXAFS spectrum (b, black) and corresponding Fourier transform (c) of neptunium sorbed on the BIOS sample; shell fits (blue). Neptunium L_III_-edge XANES spectrum (a) of Np-BIOS sample in comparison with a neptunium(IV) reference

In our study, the neptunium(V) species on the BIOS surface have also been identified as mononuclear, inner-sphere complexes (see Fig. 8 b and c), since no Np–Np interaction but a Np–Fe interaction at 3.39 Å could be detected. The structural parameters based on the shell fit analysis (Table 1) showed an average of five oxygen atoms at a Np–O_eq_ distance of 2.44 Å, two oxygen atoms at a Np–O_ax_ distance of 1.86 Å, and iron atoms at a Np–Fe distance of 3.39 Å. The Np–Fe distance is also in accord with a bidentate edge-sharing inner-sphere surface complex of the NpO_2_^+^ cation to an Fe(O,OH)_6_ octahedron (Fig. 7). Also, the XANES spectrum of the sample, which was treated with neptunium (Fig. 8a), does not show any indication for significant amount of Np(IV). The “yl-shoulder” is fully expressed at about 17,630 keV.

### Comparison of abiotic iron oxides and oxyhydroxides with BIOS samples

The radial U–O_ax_ and U–O_eq_ distances of the studied U-BIOS sample were compared with those of abiotic or synthesized iron oxides and iron oxyhydroxides determined at neutral pH. As shown in Table 2, the distances determined for U-BIOS are similar to those of abiotic ferrihydrite samples determined by Waite et al. (1994) and Rossberg et al. (2009), considering that the errors in determination of EXAFS interatomic distances and coordination numbers have an uncertainty of 0.01−0.02 Å and 10–35%, respectively. Similar distances were also measured for abiotic ferrihydrite by Ulrich et al. (2006). Recent EXAFS studies of uranium sorption on ferrihydrite by Winstanley et al. (2018) can also be used for comparison, noting however that the studies were performed under alkaline conditions and using uranium concentrations up to 1.05 mM, where precipitation of a discrete U(VI) phase was detected. However, the sorption mechanism was also described as uranium sorption to ferrihydrite via a bidentate edge-sharing inner-sphere species with carbonate forming a ternary surface complex. A splitting of the O_eq_ shell as shown for abiotic goethite, lepidocrocite, maghemite, and magnetite could only be detected in abiotic ferrihydrite samples by Waite et al. (1994). Neither the samples examined by Rossberg et al. (2009) nor the BIOS sample permits the detection of a splitting of the equatorial oxygen shell. Compared with the results reported for uranium(VI), only few EXAFS studies on the adsorption of neptunium(V) on iron oxides have been conducted at circumneutral pH, whereas no EXAFS studies have been conducted on the sorption of neptunium(V) on ferrihydrite in the near-neutral pH range. In the BIOS sample, the fitting of the first and second oxygen shell of the neptunium L_III_-edge EXAFS spectrum showed five oxygen atoms with an average Np–O_eq_ distance of 2.44 Å and two oxygen atoms at a Np–O_ax_ distance of 1.86 Å (see Table 3). The O_ax_ bond lengths are shorter than those observed for Np(V) adsorbed to hematite (Müller et al. 2015; Amayri et al. 2007) and goethite (Combes et al. 1992). According to Bots et al. (2016), this could be caused by positively charged neptunyl, forming stronger and consequently shorter bonds with negatively charged hydroxide than with the oxygen of water molecules. EXAFS studies on neptunium(V)-ferrihydrite were exclusively carried out under alkaline conditions (pH 9.5 and 11) by Bots et al. (2016). The Np–O axial bond length and the Np–O equatorial oxygen distance of the alkaline ferrihydrite samples ranged from 1.83 to 1.85 Å and 2.38 to 2.42 Å, respectively. This is in accordance with the distances we have received by fitting the EXAFS data of the BIOS sample, hence neptunium(V) sorbs in the BIOS sample also via an inner-sphere bidentate mononuclear surface complex at the ferrihydrite surface.

**Table 2:** Distances of the coordination shells of U-containing BIOS in comparison with abiotic iron oxides and iron oxyhydroxides

				Abiotic				Biogenic
	Ferrihydrite [1]	Ferrihydrite [2]	Ferrihydrite [3]	Goethite [4]	Lepidocrocite [4]	Maghemite [4]	Magnetite [4]	BIOS [5]
U–O_ax_	1.80 Å	1.80 Å	1.82 Å	1.82 Å	1.77 Å	1.83 Å	1.78 Å	1.79 Å
U–O_eq1_	2.35 Å	2.35 Å	2.38 Å	2.26 Å	2.31 Å	2.28 Å	2.26 Å	2.34 Å
U–O_eq2_				2.51 Å	2.54 Å	2.55 Å	2.52 Å	–
U–Fe	3.41 Å	3.41 Å	3.46 Å		3.44 Å			3.44 Å

[1] Waite et al. (1994)

[2] Rossberg et al. (2009);

[3] Winstanley et al. (2018)

[4] Dodge et al. (2002)

[5] This study

Radial distance error ± 0.01 Å

**Table 3:** Distances of the coordination shells of Np-containing BIOS in comparison with abiotic iron oxides and iron oxyhydroxides at circumneutral pH and for ferrihydrite at pH 9.5 and 11

	Abiotic			Biogenic		
	Lepidocrocite [1]	Hematite[2]^a^	Hematite [3]^a,b^	Goethite [4]	Ferrihydrite [5]	BIOS [6]
Np–O_ax_	1.80–1.85	1.88 Å	1.87 Å	1.85	1.83–1.85	1.86 Å
Np–O_eq1_	2.44–2.46	2.47 Å^c^/2.46 Å^d^	2.48 Å	2.51	2.38–2.42	2.44 Å
Np–O_eq2_		2.46 Å	2.84 Å	–		–
Np–Fe	3.45	3.47 Å^c^/3.73 Å^d^	3.44 Å^b^/3.73 Å^a^			3.39 Å

^a^Inert

^B^air

^c^Fit model 1

^d^Fit model 2, radial distance error ± 0.01 Å

[1] Yang et al. (2015)

[2] Müller et al. (2015)

[3] Amayri et al. (2007)

[4] Combes et al. (1992)

[5] Bots et al. (2016)

[6] This study

## Conclusions

BIOS samples, which were formed by a *G. ferruginea* strain at circumneutral pH values, were used for uranium(VI) and neptunium(V) sorption experiments. The XAS analyses showed that both uranium(VI) and neptunium(V) were sorbed by forming a bidentate edge-sharing inner-sphere sorption complex on ferrihydrite. A small fraction (5%) of uranyl also sorbed to ferrihydrite as the previously observed uranyl-carbonato complex. No influence of the organic components of BIOS on the surface speciation was found by XAS. The existence of *G. ferruginea* is of great importance for the formation of larger amounts of ferrihydrite compared with abiotic systems, resulting in numerous adsorption surface sites. The results presented here contribute to a better understanding of the potential role of BIOS in natural environments. The radionuclide adsorption observed for BIOS samples is relevant to various environmental conditions. Thus, BIOS can be regarded as an environmental barrier to prevent migration of actinides in general in radioactively contaminated sites or in the far-field of nuclear waste repositories and consequently, should be considered in developing remediation strategies and performing risk assessments.
