# Correction to: Uranium and neptunium retention mechanisms in *Gallionella ferruginea*/ferrihydrite systems for remediation purposes

**DOI:** 10.1007/s11356-021-15672-x

**Published:** 2021-08-07

**Authors:** Evelyn Krawczyk-Bärsch, Andreas C. Scheinost, André Rossberg, Katharina Müller, Frank Bok, Lotta Hallbeck, Jana Lehrich, Katja Schmeide

**Affiliations:** 1 Helmholtz-Zentrum Dresden-Rossendorf, Institute of Resource Ecology, Bautzner Landstr. 400, 01328 Dresden, Germany; 2 The Rossendorf Beamline, ESRF, F-38043 Grenoble, France; 3 Microbial Analytics Sweden AB (MICANS), SE-43535 Mölnlycke, Sweden

**Correction to: Environmental Science and Pollution Research (2021) 28:18342–18353**

10.1007/s11356-020-09563-w

The article Uranium and neptunium retention mechanisms in *Gallionella ferruginea*/ferrihydrite systems for remediation purposes written by Evelyn Krawczyk-Bärsch, Andreas C. Scheinost, André Rossberg, Katharina Müller, Frank Bok, Lotta Hallbeck, Jana Lehrich and Katja Schmeide, was originally published electronically on the publisher’s internet portal on 16 June 2020 without open access. With the author(s)’ decision to opt for Open Choice the copyright of the article changed on 22 July 2021 to © The Author(s) 2021 and the article is forthwith distributed under a Creative Commons Attribution 4.0 International License, which permits use, sharing, adaptation, distribution and reproduction in any medium or format, as long as you give appropriate credit to the original author(s) and the source, provide a link to the Creative Commons license, and indicate if changes were made. The images or other third party material in this article are included in the article’s Creative Commons license, unless indicated otherwise in a credit line to the material. If material is not included in the article’s Creative Commons license and your intended use is not permitted by statutory regulation or exceeds the permitted use, you will need to obtain permission directly from the copyright holder. To view a copy of this license, visit http://creativecommons.org/licenses/by/4.0.

The Original article has been corrected.

